# DNA copy number variations in drug reaction with eosinophilia and systemic symptoms (DRESS) identifying 2 new candidates genes

**DOI:** 10.1186/2045-7022-4-S3-O11

**Published:** 2014-07-18

**Authors:** Anne-Claire Bursztejn, Christophe Nemos, Richard Redon, Philippe Trechot, Isabelle Gastin, Annick Barbaud

**Affiliations:** 1 CHU Nancy, EA 7298 INGRES, France; 2 U954, Genetic, France; 3 UMR Inserm 1087 CNRS 6291, Institut du Thorax, France; 4 CHU Nancy, Pharmacology, France; 5 CHU Nancy, EA 7298 INGRES, Molecular Biology, France; 6 CHU Nancy, EA 7298 INGRES, Dermatology, France

## Background

Drug reaction with eosinophilia and systemic symptoms (DRESS) is a rare but severe cutaneous drug adverse reaction (SCAR) with a not well known pathogenesis. Strong genetic associations between HLA groups and drug allergy have been reported but seem to be specific for a type of SCAR, one drug and an ethnic group. Using a pangenomic approach, effective even on a small effective, by an innovating method, the Comparative Genomic Hybridization (CGH) technique the objective of this study was to determine whether some copy number variations (CNV) were associated with the occurrence of DRESS while studying only well-defined DRESS patients.

## Patients and Methods

In 18 patients with a DRESS score ≥5 according to Kardaun's criteria for Regsicar, CGH array was performed on 1M Agilent arrays. Each patient was hybridized with a sex-matching control. Five different controls (3 females and 2 males) were used. For the analysis of the CNV, we defined as significant every variation (i) that included three consecutives mer, (ii) with different limits (intersection CNV), (iii) shared by 2 patients and (iv) shared by 2 different controls. Each variation was verified in the online and in the local databases (>3000 subjects).

## Results

Among 674 identified and 23 selected CNV, 2 were shared by 4 patients. These 2 CNV were rare in the databases and contained OMIM genes, KLRC2 and CESP1. Five other patients had CNV at the CES1 locus and 2 other patients at the KLRC2 locus. CNV at the CES1 locus were either deletion of the gene or amplification of the pseudogene. Every CNV at the KLRC2 locus were amplifications.

## Discussion

The CESP1 gene is the pseudogene of the gene encoding carboxylesterase 1 that is one of the major enzyme for drug metabolism. Both genes are contigus. The pseudogene could be inhibitor of the gene. The KLRC2 gene encodes a lectin type receptor from the NK receptor family that activates the cytotoxic answer. It is associated with high granulysin expression, which is implicated in SCAR. A genetically disturbed drug metabolism could induce drug intolerance. This phenomenon associated with an amplified cytotoxic answer (after herpes virus reactivation?) could explain most symptoms seen during DRESS.

## Conclusion

This study is, to our knowledge, the first one to use CGH array to identify candidate gene in SCAR, could identified 2 interesting genes. These data have to be confirmed by replication studies and functional assays.

